# Assessment practices of peripheral venous catheters use: results of a multicentre observational study in France in 2010

**DOI:** 10.1186/1753-6561-5-S6-P53

**Published:** 2011-06-29

**Authors:** D Verjat-Trannoy, D Thillard, F Daniel, M Aupée, C Laland, L Simon, M Giard, C Rabaud, P Astagneau, and the members of the GREPHH

**Affiliations:** 1 CCLIN Paris-Nord, Paris, France; 2 CCLIN Ouest, Rennes, France; 3 CCLIN Sud-Ouest, Poitiers, France; 4 CCLIN Est, Nancy, France; 5 CCLIN Sud-Est, Lyon, France

## Introduction / objectives

The frequent use of peripheral venous catheters (PVC) generate a significant number of infections. Since 2005, a standard recommendation edited by the French Society for Hospital Hygiene has been promoted in healthcare facilities (HCF). In 2009, the French Group for Evaluation of Practices concerning Hospital Hygiene (GREPHH) has provided an evaluation tool for PVC practices.

The objective was to evaluate practices during utilisation of PVC in healthcare facilities (HCF).

## Methods

In 2009, the five regional coordinating centres for nosocomial infection control (CCLIN) launched an observational practice study based on the GREPHH evaluation tool in all volunteer HCF in France. Evaluation criteria included protocol of cares, practices of insertion and handling of venous lines, traceability and duration of the device. Proportion of practice breaches were analyzed considering category of hospital and speciality.

## Results

The study was conducted in 920 HCF and 8254 clinical wards. Standard protocols were present in 98% of the HCF: 34% of them complied with the 10 quality standards defined. The recommended “4 times” procedure represents 46% of adult skin preparation. Gloves were used just before insertion in 63% of PVC. The injection site was correctly disinfected in 60% of venous lines handlings. The insertion date was traced for 79% of the PVC, better than the daily clinical monitoring (70%). The duration period was maximum 4 days for 92% of the adult PVC.

## Conclusion

This study highlighted conformity to standard practices of PVC utilization is still poor and should merit more promotion efforts, especially for skin disinfection.

## Disclosure of interest

None declared.

